# Associations between STAT3 rs744166 Polymorphisms and Susceptibility to Ulcerative Colitis and Crohn's Disease: A Meta-Analysis

**DOI:** 10.1371/journal.pone.0109625

**Published:** 2014-10-06

**Authors:** Jixiang Zhang, Jianhong Wu, Xiulan Peng, Jia Song, Jun Wang, Weiguo Dong

**Affiliations:** 1 Department of Gastroenterology, Renmin Hospital of Wuhan University, Wuhan, Hubei Province, China; 2 Wuhan medical treatment center, Wuhan, Hubei Province, China; 3 Department of Oncology, The Fifth Hospital of Wuhan, Wuhan, Hubei Province, China; University of Aberdeen, United Kingdom

## Abstract

**Background:**

Many studies have investigated the associations between the signal transducer and activator of transcription 3 (STAT3) in the susceptibility to ulcerative colitis (UC) and Crohn's disease (CD). However, the results remain inconsistent. This meta-analysis determined the risk of STAT3 rs744166 polymorphism-conferred UC and CD susceptibility.

**Materials and Methods:**

Electronic databases, including PubMed, EMBASE and the Cochrane Library, were searched for all eligible studies that evaluated the association between STAT3 rs744166 polymorphisms with UC and CD risk up to August 21, 2014. The pooled odds ratios (ORs) and 95% confidence intervals (95% CIs) were calculated using fixed- or random-effects models.

**Results:**

Twelve studies containing 10298 patients with CD, 4244 patients with UC and 11191 controls were included in this meta-analysis. The results indicated that the STAT3 rs744166 polymorphism was associated with CD and UC susceptibility (CD: GA+AA vs. GG, OR = 1.20, 95%CI, 1.11–1.30, *I* ^2^ = 0%, *P*_unadjusted_<0.00001, *P*_Bonferroni_<0.00005, *P*_FDR_<0.00001; UC: GA+AA vs. GG, OR = 1.21, 95%CI, 1.08–1.36, *I* ^2^ = 1%, *P*_unadjusted_ = 0.001, *P*_Bonferroni_ = 0.005, *P*_FDR_ = 0.00125). In subgroup analyses by ethnicity, the significant association was found only among Caucasians. However, when grouped by age of onset, positive associations were found both among adults and children. In addition, when stratified by study design and genotyping methods, the risk of CD was significantly associated with the STAT3 rs744166 polymorphism in hospital-based and population-based groups and in SNP Array and SNPlex groups. For UC, significant associations were also found in population-based, PCR-RFLP and SNPlex groups. Moreover, these findings were sufficiently robust to withstand the Bonferroni correction and false discovery rate (FDR).

**Conclusion:**

This meta-analysis indicates that carriers of the STAT3 rs744166 ‘A’ allele have a significantly greater risk of CD and UC, especially among Caucasians.

## Introduction

As a non-specific, intestinal inflammatory disorder, inflammatory bowel disease (IBD) consists of ulcerative colitis (UC) and Crohn's disease (CD) clinically. The inflammation in UC only involves the mucosal and submucosal layers of the rectum and colon and is continuous. In contrast, in CD, the inflammation may affect any part of the digestive tract and is intermittent [1], [2]. With the increasing incidence and prevalence of IBD, there are more studies researching the risk factors and pathogenesis [3]. How genetic factors affect the occurrence and development of IBD has drawn increasing attention [4]–[6]. Until now, approximately 100 IBD-susceptibility loci, including 70 loci specific to CD and 47 specific to UC, have been identified. Their functions include microbe recognition, lymphocyte activation, cytokine signaling, and intestinal epithelial defense [7]–[9]. Though IBD-susceptibility loci, such as nucleotide oligomerization domain 2 (NOD2), immunity-related GTPase family M (IRGM), interleukin 23 receptor (IL23R) and autophagy related 16-like 1 (ATG16L1), have been previously identified, their roles in the incidence of IBD remain controversial [10]–[13].

The family of signal transducers and activators of transcription (STATs) contains many intracellular effector molecules of cytokine-modulated signaling, which could affect the development of the immune system and hematopoiesis. After activation through tyrosine phosphorylation, STATs combine to form dimers and then are transported to the nucleus to induce transcription. As an important member of the STAT family, STAT3 can be activated by IL-6 and IL-23 after combining with IL-1b, TGF- b, and RoRct. STAT3 also plays an important role in T helper 17 (Th17) formation [14], though it can also be activated by IL-10. IL-10 activation can then affect the function of Tregs, including the ability to inhibit coinciding pathogenic Th17 responses [15]. Because STAT3 can interact with many IBD-related cytokines and molecules, it plays an important role in the pathogenesis of IBD.

Recently, a number of studies have investigated the association between the STAT3 rs744166 polymorphism and UC and CD susceptibility, but the results remain inconclusive. Though a meta-analysis [16] has already discussed this association and the conclusion is convincing, the impact of ethnicity, age of onset and study design on the risk of STAT3 rs744166 polymorphism-conferred UC and CD susceptibility is unclear. Therefore, we conducted a meta-analysis of the previously published studies involving STAT3 rs744166 polymorphism and UC and CD susceptibility and these potential influential factors to clarify the impact of this polymorphism.

## Materials and Methods

### Search strategy

We searched the electronic databases, including PubMed, EMBASE and the Cochrane Library, for all eligible studies that evaluated the association between the STAT3 rs744166 polymorphism and UC and CD risk up to August 21, 2014. The relevant studies were identified using the following key words and subject terms: “inflammatory bowel disease” or “IBD”; “ulcerative colitis” or “UC”; “Crohn's disease” or “CD”; “signal transducers and activators of transcription 3” or “STAT3”; and “genetic polymorphism” or “polymorphism” or “variant”. Additional studies were identified by searching the reference lists of the identified studies. The search was restricted to humans and did not have a language limitation.

### Inclusion and exclusion criteria

The studies were included the meta-analysis if they met the following criteria: (1) case-control study design; (2) investigated the association between UC and/or CD with the STAT3 rs744166 polymorphism; (3) controls were from a healthy population or were patients without diseases related to IBD; and (4) had detailed genotype frequencies of the cases and controls (or could be calculated from the article text). Studies were excluded if: (1) the research did not study the STAT3 rs744166 polymorphism; (2) no report of genotype frequency; (3) case studies, case reports and review articles and (4) no control group.

### Data extraction

The following information regarding each eligible trial was extracted by two investigators independently: the first author's name, year of publication, country of origin, ethnicity of study population, genotype method, number of cases and controls and H-W equilibrium in controls. Any encountered discrepancies were resolved by consensus.

### Statistical analysis

The meta-analysis was performed using the Cochrane Collaboration RevMan 5.1 and STATA package version 12.0 (Stata Corporation, College Station, TX, USA). The pooled odds ratios (OR) and 95% confidence intervals (CI) were calculated to evaluate the association between the STAT3 rs744166 polymorphisms and UC and CD risk. In addition, subgroup analyses were performed based on ethnicity and study design when adequate data were available. A χ^2^-test based on the Q statistic was performed to assess the between-study heterogeneity. When *I* ^2^>50% and *P*<0.1, the heterogeneity was considered to be significant, and the random effects model was used to analyze the data. The fixed effects model was chosen for homogeneous data. Egger's test was used to assess the publication bias. HWE was examined with the χ^2^ test. *P*<0.05 was considered to be significant. To adjust for multiple comparisons, the Bonferroni correction and false discovery rate (FDR) were applied. The power of the meta-analysis for each polymorphism to detect an effect size was estimated according to the method recommended by Hedges and Pigott [17] with a significance value of 0.05.

## Results

### Studies included in the meta-analysis

**Figure 1:**
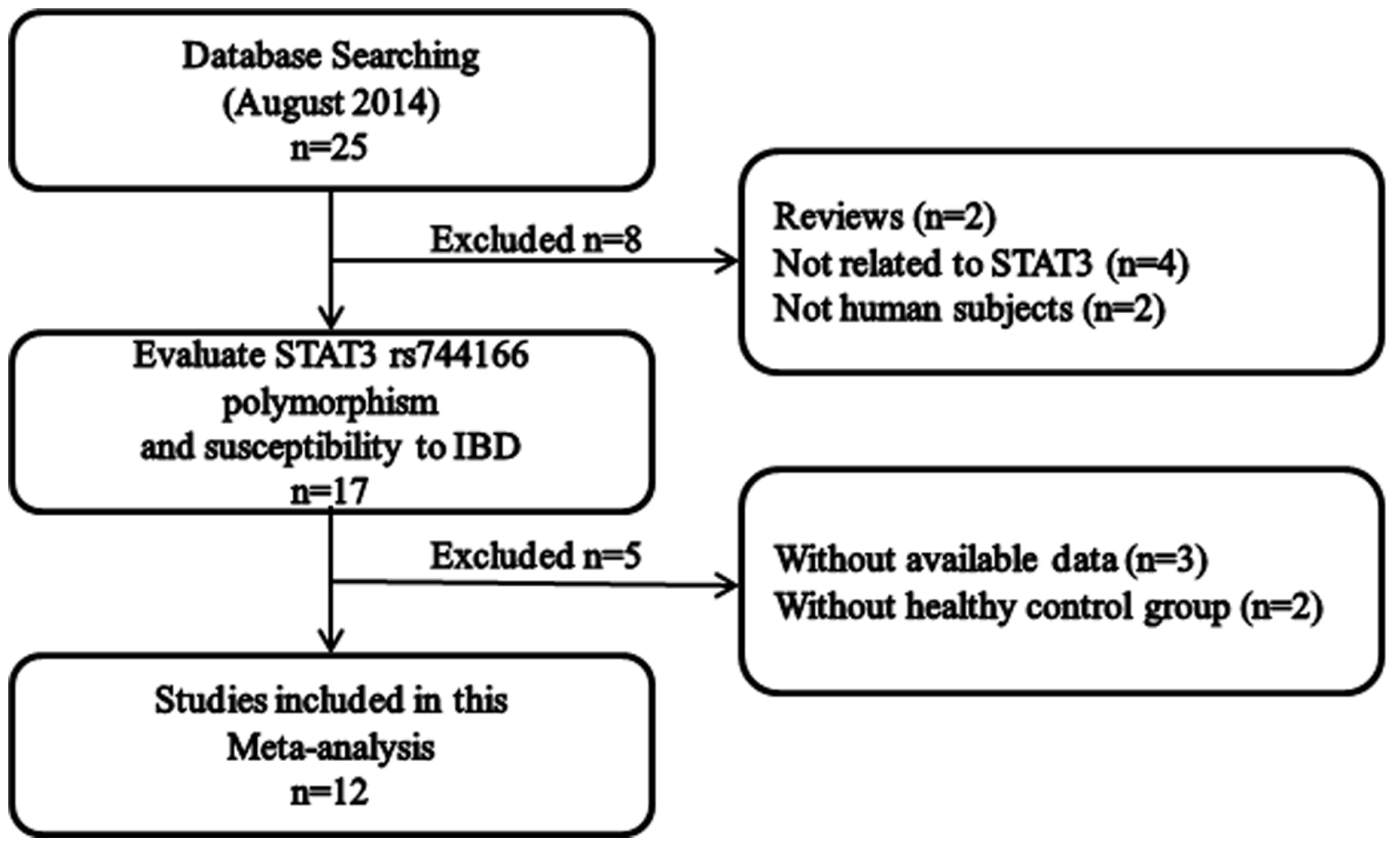
The screening process of studies.

Following the searching strategy, 25 potentially relevant studies were retrieved. According to the inclusion criteria, 12 studies [18]–[29] with full-text were included in this meta-analysis, and 13 studies were excluded (Fig 1). Eleven studies [18]–[28] reported the association between STAT3 rs744166 polymorphisms and CD. And five studies [19], [20], [23], [27], [29] examined the associations between STAT3 rs744166 polymorphisms and UC. Four studies [19], [20], [23], [27] examined the association between STAT3 rs744166 polymorphisms and both CD and UC (Table 1). The distribution of genotypes in the controls was consistent with the Hardy-Weinberg equilibrium for all selected studies.

**Table 1:** Characteristics of the studies included in the meta-analysis.

Study	Year	Country	Ethnicity	Study Design	Age of Onset	Genotyping Method	Sample Size		
							CD	UC	Control
Jung [18]	2012	France	Multi-ethnicity	HB	Adult	SNP Array	798	—	960
Waterman [19]	2011	Canada	Multi-ethnicity	HB	Multi-age	SNP Array	1140	1230	1057
Franke [20]	2008	Germany	Caucasian	PB	Multi-age	SNPlex	1845	1099	1791
Amre [21]	2010	Canada	Caucasian	HB	Child	Sequenom platform	406	—	415
Peter [22]	2011	USA	Caucasian	PB	Multi-age	Taqman	503	—	369
Polgar [23]	2012	Hungary	Caucasian	PB	Adult	PCR-RFLP	309	307	496
Danoy(Phase1) [24]	2010	Australia	Caucasian	PB	Multi-age	SNP Array	1230	—	1295
Danoy(Phase2) [24]	2010	Australia	Caucasian	PB	Multi-age	SNPlex	1545	—	920
Laukens [25]	2010	Belgium	Caucasian	HB	Adult	SNP Array	1071	—	693
Ferguson [26]	2010	New Zealand	Caucasian	PB	Multi-age	Taqman	302	—	382
Cénit [27]	2010	Spain	Caucasian	HB	Adult	Taqman	394	442	1692
Henckaerts [28]	2009	Belgium	Caucasian	HB	Multi-age	PCR-RFLP	755	—	344
Franke [29]	2008	Germany	Multi-ethnicity	HB	Multi-age	SNP Array	—	1166	777

Rs744166 polymorphism was in the Hardy-Weinberg equilibrium for controls.

### Associations between STAT3 rs744166 polymorphisms and CD risk

A summary of the meta-analysis findings concerning the associations between the STAT3 rs744166 polymorphisms and CD is shown in Tables 2 and 3.

**Table 2:** Pooled analysis of the association between the STAT3 rs744166 polymorphisms with the risk of CD and UC.

Disease	N	Comparison	Test of Association		Bonferroni	FDR	Test of Heterogeneity		Publication Bias *P*-value
			OR (95% CI)	*P*-value			*P*-value	*I* ^2^ (%)	
CD	11	GA vs. GG	1.14(1.05–1.24)	0.00100	0.00500	0.00100	0.850	0	0.188
		AA vs. GG	1.29(1.19–1.40)	<0.00001	<0.00005	<0.00001	0.230	22	0.292
		GA+AA vs. GG	1.20(1.11–1.30)	<0.00001	<0.00005	<0.00001	0.510	0	0.240
		AA vs. GG+GA	1.17(1.10–1.24)	<0.00001	<0.00005	<0.00001	0.430	1	0.399
		A vs. G	1.13(1.09–1.18)	<0.00001	<0.00005	<0.00001	0.210	24	0.284
UC	5	GA vs. GG	1.14(1.01–1.29)	0.04000	0.20000	0.04000	0.780	0	0.508
		AA vs. GG	1.31(1.16–1.49)	<0.00010	<0.00050	<0.00017	0.160	40	0.825
		GA+AA vs. GG	1.21(1.08–1.36)	0.00100	0.00500	0.00125	0.400	1	0.647
		AA vs. GG+GA	1.19(1.09–1.30)	<0.00010	<0.00050	<0.00017	0.190	34	0.776
		A vs. G	1.15(1.08–1.22)	<0.00001	<0.00005	<0.00005	0.110	47	0.689

Bonferroni, *P*-value in Bonferroni testing; FDR, *P*-value in false discovery rate.

**Table 3:** Subgroup analysis of the association between the STAT3 rs744166 polymorphisms and the risk of CD.

Basis for grouping	Comparison	Subgroup	Test of Association		Bonferroni	FDR	Test of Heterogeneity		Heterogeneity Between Subgroups	
			OR (95% CI)	*P*-value			*P*-value	*I* ^2^ (%)	*P*-value	*I* ^2^ (%)
Ethnicity	GA+AA vs. GG	Caucasian	1.24(1.14–1.35)	<0.00001	<0.00005	<0.00001	0.620	0	0.080	66.6
		Multi-ethnic	1.05(0.89–1.25)	0.55000	1.00000	0.68750	0.950	0		
	A vs. G	Caucasian	1.16(1.11–1.21)	<0.00001	<0.00005	<0.00001	0.380	6	0.030	79.1
		Multi-ethnic	1.04(0.95–1.13)	0.44000	1.00000	0.68750	0.910	0		
Study Design	GA+AA vs. GG	HB	1.21(1.08–1.36)	0.00100	0.00500	0.00167	0.270	22	0.860	0
		PB	1.19(1.08–1.32)	0.00060	0.00300	0.00075	0.580	0		
	A vs. G	HB	1.12(1.06–1.19)	0.00020	0.00100	0.00050	0.200	32	0.640	0
		PB	1.14(1.08–1.21)	<0.00001	<0.00005	0.00003	0.230	27		
Age of Onset	GA+AA vs. GG	Child	1.41(0.98–2.02)	0.06000	0.30000	0.07500	—	—	0.670	0
		Adult	1.18(1.02–1.36)	0.03000	0.15000	0.05000	0.680	0		
		Multi-age	1.20(1.09–1.31)	0.00010	0.00050	0.00013	0.250	24		
	A vs. G	Child	1.27(1.05–1.55)	0.02000	0.10000	0.05000	—	—	0.400	0
		Adult	1.10(1.02–1.19)	0.01000	0.05000	0.02500	0.640	0		
		Multi-age	1.14(1.08–1.19)	<0.00001	<0.00005	<0.00002	0.090	45		
Genotyping Methods	GA+AA vs. GG	PCR-RFLP	1.37(1.05–1.78)	0.02000	0.10000	0.03333	0.160	50	0.660	0
		SNP Array	1.15(1.03–1.29)	0.02000	0.10000	0.02500	0.550	0		
		Taqman	1.25(1.02–1.55)	0.04000	0.20000	0.06667	0.530	0		
		SNPlex	1.18(1.03–1.35)	0.02000	0.10000	0.02500	0.130	57		
	A vs. G	PCR-RFLP	1.19(1.04–1.36)	0.01000	0.05000	0.02500	0.200	39	0.790	0
		SNP Array	1.11(1.04–1.18)	0.00200	0.01000	0.00500	0.280	22		
		Taqman	1.14(1.03–1.27)	0.02000	0.10000	0.05000	0.200	37		
		SNPlex	1.14(1.06–1.22)	0.00070	0.00350	0.00250	0.070	69		
Number of patients	GA+AA vs. GG	Small size (<1000)	1.24(1.02–1.50)	0.03000	0.15000	0.03750	0.580	0	0.720	0
		Moderate size (1000–2500)	1.23(1.10–1.36)	0.00010	0.00050	0.00017	0.250	24		
		Large size (>2500)	1.15(1.00–1.31)	0.04000	0.20000	0.05000	0.320	0		
	A vs. G	Small size (<1000)	1.17(1.06–1.30)	0.00200	0.01000	0.00833	0.220	31	0.720	0
		Moderate size (1000–2500)	1.13(1.07–1.20)	<0.00010	<0.00050	<0.00017	0.150	39		
		Large size (>2500)	1.11(1.04–1.20)	0.00300	0.01500	0.01167	0.270	19		

HB, hospital-based; PB, population-based; Bonferroni, *P*-value in Bonferroni testing; FDR, *P*-value in false discovery rate.

**Figure 2:**
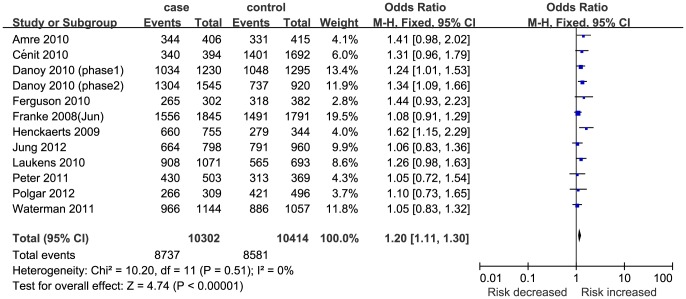
Meta-analysis of the association between STAT3 rs744166 polymorphism and CD for GA+AA vs. GG.

Eleven studies [18]–[28], which were comprised of 10302 cases and 10414 controls, reported an association between STAT3 rs744166 polymorphism and CD susceptibility. The STAT3 rs744166 polymorphism was significantly associated with CD susceptibility (Table 2 and Fig 2). Even with the Bonferroni correction and FDR, the result remained reliable.

**Figure 3:**
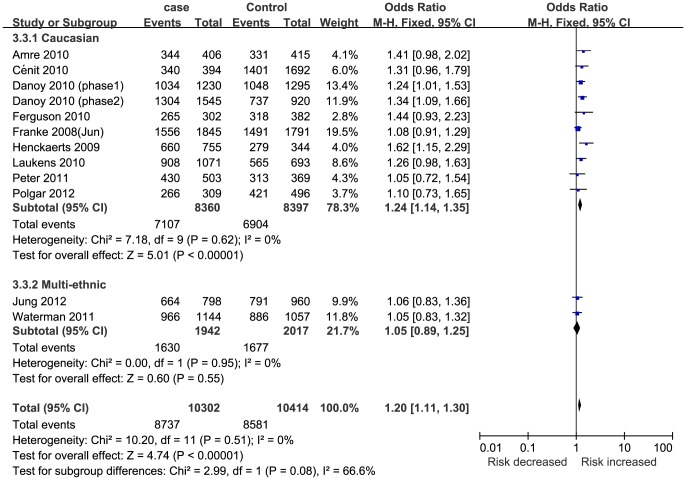
Subgroup analysis of the association between STAT3 rs744166 polymorphism and CD by ethnicity for GA+AA vs. GG.

The patients in nine studies [20]–[28] were Caucasian, and two studies [18], [19] consisted of various ethnicities. According to the subgroup analysis by ethnicity, STAT3 rs744166 polymorphisms were significantly associated with CD susceptibility in Caucasian but not in multi-ethnic group (Table 3 and Fig 3). Six studies [18], [19], [21], [25], [27], [28] were hospital-based, and five studies [20], [22]–[24], [26] were population-based. According to the subgroup analysis by study design, significant associations were found between STAT3 rs744166 polymorphisms and CD susceptibility in both the hospital-based and population-based groups (Table 3). In addition, the patients in one study [21] were children; four studies [18], [23], [25], [27] included adults; and six studies [19], [20], [22], [24], [26], [28] included both children and adults. The subgroup analysis by age of onset indicated that STAT3 rs744166 polymorphism was significantly associated with CD susceptibility only in the multi-age group (Table 3). The genotyping method in two studies [23], [28] was PCR-RFLP; four studies [18], [19], [24], [25] used the SNP Array; three studies [22], [26], [27] used Taqman; and two studies [20], [24] used SNPlex. The subgroup analysis by genotyping method indicated that, except for Taqman, STAT3 rs744166 polymorphism was significantly associated with CD susceptibility with the other genotyping methods (Table 3). Moreover, the subgroup analysis according to the number of patients (four studies [21]–[23], [26] had <1000 patients; six studies [18], [19], [24], [25], [27], [28] had 1000–2500 patients; and two studies [20], [24] had>2500 patients) indicated that significant associations were found in each group. The results with the Bonferroni correction and FDR applied to each subgroup analysis indicated that the results of each subgroup analysis (with respect to ethnicity, study design, age of onset, genotyping method and number of patients) were stable and reliable.

### Associations between STAT3 rs744166 polymorphism and UC risk

A summary of the meta-analysis findings concerning the associations between STAT3 rs744166 polymorphism and UC risk is shown in Table 2 and Table 4.

**Table 4:** Subgroup analysis of the association between the STAT3 rs744166 polymorphisms and the risk of UC.

Basis for grouping	Comparison	Subgroup	Test of Association		Bonferroni	FDR	Test of Heterogeneity		Heterogeneity Between Subgroups	
			OR (95% CI)	*P*-value			*P*-value	*I* ^2^ (%)	*P*-value	*I* ^2^ (%)
Ethnicity	GA+AA vs. GG	Caucasian	1.33(1.13–1.56)	0.00050	0.00250	0.00063	0.800	0	0.1000	63.8
		Multi-ethnic	1.09(0.92–1.29)	0.32000	1.00000	0.40000	0.360	0		
	A vs. G	Caucasian	1.22(1.13–1.33)	<0.00001	<0.00005	<0.000023	0.580	0	0.020	80.6
		Multi-ethnic	1.06(0.97–1.16)	0.17000	0.85000	0.36667	0.240	27		
Study Design	GA+AA vs. GG	HB	1.14(0.98–1.32)	0.08000	0.40000	0.10000	0.400	0	0.180	44.0
		PB	1.34(1.11–1.63)	0.00300	0.01500	0.00017	0.520	0		
	A vs. G	HB	1.09(1.01–1.18)	0.03000	0.15000	0.07500	0.280	22	0.040	76.9
		PB	1.24(1.13–1.37)	<0.00001	<0.00005	<0.00005	0.400	0		
Age of Onset	GA+AA vs. GG	Adult	1.37(1.07–1.75)	0.01000	0.05000	0.01250	0.540	0	0.280	14.8
		Multi-age	1.17(1.02–1.34)	0.02000	0.10000	0.02500	0.290	19		
	A vs. G	Adult	1.23(1.09–1.39)	0.00100	0.00500	0.00500	0.300	7	0.200	39.0
		Multi-age	1.12(1.05–1.20)	0.00100	0.00500	0.00500	0.090	59		
Genotyping Methods	GA+AA vs. GG	PCR-RFLP	1.53(0.99–2.38)	0.06000	0.30000	0.07500	—	—	0.360	6.5
		SNP Array	1.09(0.92–1.29)	0.32000	1.00000	0.40000	0.360	0		
		Taqman	1.30(0.96–1.75)	0.09000	0.45000	0.12500	—	—		
		SNPlex	1.30(1.05–1.61)	0.02000	0.10000	0.02500	—	—		
	A vs. G	PCR-RFLP	1.35(1.09–1.67)	0.00600	0.03000	0.01667	—	—	0.140	45.0
		SNP Array	1.06(0.96–1.18)	0.25000	1.00000	0.40000	0.240	27		
		Taqman	1.17(1.01–1.37)	0.04000	0.20000	0.10000	—	—		
		SNPlex	1.22(1.09–1.36)	0.00040	0.00002	0.00167	—	—		
Number of patients	GA+AA vs. GG	Small size (<1000)	1.53(0.99–2.38)	0.06000	0.30000	0.01667	—	—	0.280	13.7
		Large size (>1000)	1.19(1.06–1.34)	0.00500	0.02500	0.00625	0.410	0		
	A vs. G	Small size (<1000)	1.35(1.09–1.67)	0.00600	0.03000	0.01667	—	—	0.120	57.8
		Large size (>1000)	1.13(1.04–1.23)	0.00500	0.02500	0.00625	0.160	42		

HB, hospital-based; PB, population-based; Bonferroni, *P*-value in Bonferroni testing; FDR, *P*-value in false discovery rate.

**Figure 4:**
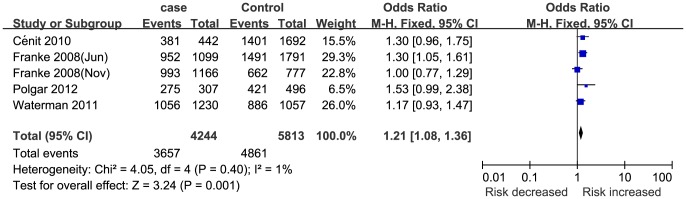
Meta-analysis of the association between STAT3 rs744166 polymorphism and UC for GA+AA vs. GG.

Five studies [19], [20], [23], [27], [29] (4244 cases and 5813 controls) reported an association between STAT3 rs744166 polymorphism and UC susceptibility. In this meta-analysis, STAT3 rs744166 polymorphism was significantly associated with UC susceptibility (Table 2 and Fig 4). This finding was sufficiently robust to withstand the Bonferroni correction and FDR.

**Figure 5:**
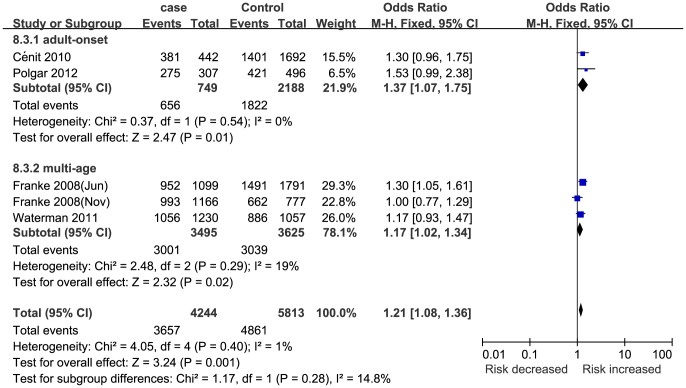
Subgroup analysis of the association between STAT3 rs744166 polymorphism and UC by age of onset for GA+AA vs. GG.

The patients in three studies [20], [23], [27] were Caucasian, and two studies [19], [29] consisted of multiple ethnicities. According to the subgroup analysis by ethnicity, STAT3 rs744166 polymorphism was significantly associated with UC susceptibility in the Caucasian group but not in the multi-ethnic group (Table 4). Three studies [19], [27], [29] were hospital-based, and two studies [20], [23] were population-based. According to the subgroup analysis by study design, significant associations were found between STAT3 rs744166 polymorphism and UC susceptibility in the population-based group but not in the hospital-based group (Table 4). The patients in two studies [23], [27] were adults, and three studies [19], [20], [29] contained both children and adults. The subgroup analysis by age of onset indicated that STAT3 rs744166 polymorphism was significantly associated with UC susceptibility in both the adult and multi-age groups (Table 4 and Fig 5). Meanwhile, the genotyping method in two studies [19], [29] was a SNP Array, and the other three studies [20], [23], [27] used other methods, including TaqMan, SNPlex and PCR-RFLP. The subgroup analysis by genotyping method indicated that STAT3 rs744166 polymorphism was significantly associated with UC susceptibility in the SNPlex group but not in other genotyping method groups (Table 4). Additionally, the subgroup analysis by number of patients (one study [23] had <1000 patients; four studies [19], [20], [27], [29] had>1000 patients) indicated that significant associations were found in both groups. In addition, the results of each subgroup analysis after the Bonferroni correction and FDR also support the findings involving ethnicity, study design, age of onset, genotyping method and number of patients.

### Test of heterogeneity and publication bias

The heterogeneity of the included studies in regards to each polymorphism is presented in Table 2, Table 3 and Table 4. There was no significant heterogeneity in the meta-analysis pertaining to the associations between STAT3 rs744166 polymorphism and CD and UC susceptibility.

Egger's test was used to assess for publication bias. According to Egger's Test, we found no evidence of publication bias (Table 2).

## Discussion

IBD has no identified cause and is a chronic, relapsing, intestinal inflammatory disease that consists of UC and CD. Altogether, IBD is associated with the complex interactions between genetic and environmental factors that cause an intestinal inflammatory response [30], [31]. By altering protein and cytokine function (and then the individual's susceptibility to IBD), gene variants may have an important role in the pathogenesis of this common disease [32], [33]. To date, a number of IBD-related genes have been identified. Among these genes, STAT3 has been highlighted.

The activation of STAT3, which plays an important role in the inflammatory response, in intestinal epithelial cells and myeloid cells promotes the development of colitis-associated cancer and influences the anti-inflammatory effects of IL-10 [34]–[37]. Furthermore, in CD4^+^ T cells, STAT3 activation affects the blockade of IL-6 and the differentiation of TH17 effector lymphocytes [38]. To date, several loci located in STAT3 have been identified and the associations between them and IBD susceptibility have been evaluated in some studies. However, the results remain inconsistent and inconclusive. Thus, performing a meta-analysis to evaluate the associations between STAT3 rs744166 polymorphism and IBD is necessary.

In our meta-analysis, eleven studies [18]–[28] (with 10302 cases and 10414 controls) reported an association between STAT3 rs744166 polymorphism and CD susceptibility. Five studies [19], [20], [23], [27], [29] (with 4244 cases and 5813 controls) reported an association between STAT3 rs744166 polymorphism and UC susceptibility. The first major finding of this meta-analysis was that “A” allele carriers have a higher risk of developing CD and UC. The mechanism of how the “A” allele of the STAT3 rs744166 gene influences the susceptibility to CD and UC is still unclear. One possibility is that the “A” allele changes the interaction between STAT3 and other inflammation-related signaling molecules upstream and downstream. The second major finding of this meta-analysis was that Caucasian “A” allele carriers are more likely to develop CD and UC than multi-ethnic groups. As we know, many diseases are affected by genetic differences or environmental features. As non-specific, intestinal inflammatory disorders, CD and UC can be influenced by ethnicity, diet, living habit and environment. However, the mechanisms by which these factors function should be discussed in future studies. The third finding of this meta-analysis was that “A” allele carriers in the population-based group (but not in the hospital-based group) were susceptible to UC. In addition, STAT3 rs744166 polymorphism was significantly associated with CD susceptibility only in the multi-age group, and STAT3 rs744166 polymorphism was significantly associated with UC susceptibility both in the adult and multi-age groups. Because the initial symptoms of CD and UC may be mild, and patients are usually diagnosed by a physician many years later when the symptoms become obvious. Therefore, the impact of the age of onset on the association between STAT3 rs744166 polymorphism and CD susceptibility should be interpreted cautiously and confirmed by more studies. The last finding was that, except for Taqman, STAT3 rs744166 polymorphism was significantly associated with CD susceptibility with other genotyping methods. Except for the SNP Array group, STAT3 rs744166 polymorphism was significantly associated with UC susceptibility with other genotyping methods.

Two insurmountable limitations of this meta-analysis should be addressed. First, several relevant studies could not be included due to incomplete raw data. Second, because not all of the necessary information could be obtained, the relevant stratifications could not be made for many studies.

In conclusion, this meta-analysis suggests that STAT3 rs744166 polymorphisms may increase the risk for CD and UC, especially among Caucasians. Gene-gene and gene-environment interactions should be investigated in the future.

## Supporting Information

Checklist S1 **PRISMA Checklist.**
(DOC)

Checklist S2 **MOOSE Checklist.**
(DOC)
