# Supplementary material for: Associations between STAT3 rs744166 Polymorphisms and Susceptibility to Ulcerative Colitis and Crohn's Disease: A Meta-Analysis
Source: PLoS One. 2014 Oct 6;9(10):e109625. doi: 10.1371/journal.pone.0109625 (PMC4186844; doi:10.1371/journal.pone.0109625)
Supplement: Checklist S2 — MOOSE Checklist. (DOC) [file pone.0109625.s002.doc]

**MOOSE Checklist**

**Associations Between STAT3 rs744166 Polymorphisms and Susceptibility to**

**Ulcerative Colitis and Crohn's Disease: A Meta-analysis**

Ji-Xiang Zhang, Jia Song, Jun Wang, Wei-Guo Dong

Department of gastroenterology, Renmin Hospital of Wuhan University, Wuhan 430060, Hubei Province, China.

Corresponding Author: Weiguo Dong

Department of Gastroenterology, Renmin Hospital of Wuhan University, 238 Jiefang Road, Wuhan 430060, Hubei Province, China.

E-mail: dwg@whu.edu.cn

Phone: 86-27-88041911

Fax: 86-27-88042292

1. **Reporting of background should include**

**Problem definition:** STAT3 rs744166 Polymorphisms and Ulcerative Colitis and Crohn's Disease risk

**Hypothesis statement:** STAT3 rs744166 Polymorphisms contribute to increasing Ulcerative Colitis and Crohn's Disease risk

**Description of study outcome:** This meta-analysis indicated that STAT3 rs744166 polymorphism was a risk factor for CD and UC risk, especially in Caucasian. The differences in age of onset, study design and genotyping method did not influence the associations obviously.

**Type of exposure or intervention used:** STAT3 rs744166 Polymorphisms

**Type of study designs used:** Meta-analysis

**Study population:** Ulcerative Colitis or Crohn's Disease patients and healthy controls

1. **Reporting of search strategy should include**

**Qualifications of searchers:** Jixiang Zhang and Jun Wang

**Search strategy, including time period include in the synthesis and keywords:**

PubMed from 1965 –February 2014

Cochrane Library from 1997 –February 2014

EMBASE from 1985 –February 2014

STAT3; polymorphisms; Ulcerative Colitis; Crohn’s disease; Meta-analysis

**Effort to include all available studies, including contact with authors:** Yes

**Databases and registries searched:** PubMed, EMBASE, Cochrane Library

**Search software used, name and version, including special features used:** We did not employ any search software. EndNote was used to merge retrieved citations and eliminate duplications

**Use of hand searching:** Yes

**List of citations located and those excluded, including justification:** Figure 1 and Table 1

**Method of addressing articles published in languages other than English:** Translation software

**Method of handing abstracts and unpublished studies:** No unpublished studies were observed.

**Description of any contact with authors:** None

1. **Reporting of methods should include**

**Description of relevance or appropriateness of studies assembled for assessing the hypothesis to be tested:** Table 1

**Rationale for the selection and coding of data:** (1) in a case-control study design; (2) the study investigated the association between UC and/or CD with STAT3 rs744166 polymorphism; (3) controls of included studies were from a healthy population or were subjects without diseases related to IBD and (4) had detailed genotype frequency of cases and controls or could be calculated from the article text

**Documentation of how data were classified and coded:** Two investigators (Zhang and Wang) used a standard protocol and data-collection form. They discussed with Pro. Dong, then decide the data.

**Assessment of confounding:** No restricted for the analysis. Conducted sensitivity analyses by eliminating the study that was not in HWE.

**Assessment of study quality, including binding of quality assessors; stratification or regression on possible predictors of study results:** The results of sensitivity analyses were very stable.

**Assessment of heterogeneity:** The chi-square-based *Q*-test and *I^2^* test

**Description of statistical methods in sufficient detail to be replicated:** A χ^2^-test-based Q statistic test was performed to assess the between-study heterogeneity. We also quantified the effect of heterogeneity by *I*^2^ test. When a significant Q test (*P*<0.05) or *I*^2^ >50% indicated heterogeneity across studies, the random effects model was used, or else the fixed effects model was used.

**Provision of appropriate tables and graphics:** We included the terms used for database search, 1 flow chart, 4 summary tables, 4 forest plots of all studies.

1. **Reporting of results should include**

**Graphic summarizing individual study estimates and overall estimate:** Table 2, Table 3, Table 4, Figure 2, Figure 3, Figure 4, Figure 5

**Table giving descriptive information for each study included:** Table 2, Table 3, Table 4

**Results of sensitivity testing:** Table 2, Table 3, Table 4

**Indication of statistical uncertainty of findings:** 95% confidence intervals were presented with all summary estimates, *P* values and results of sensitivity analyses.

1. **Reporting of discussion should include**

**Quantitative assessment of bias:** Sensitivity analyses indicate this non-significant association was stable.

**Justification for exclusion:** We excluded studies that had used different exposure or outcome assessment for the comparison groups, or no control group.

**Assessment of quality of included studies:** We discussed the results of the sensitivity analyses.

1. **Reporting of conclusions should include**

**Consideration of alternative explanations for observed results:** We discussed that potential unmeasured confounders such as differences of ethnicity, the source of controls, particular study, life style, environment background and other unknown factors may be the source of heterogeneity.

**Generalization of the conclusions:** This meta-analysis indicated that STAT3 rs744166 polymorphism was a risk factor for CD and UC risk, especially in Caucasian. The differences in age of onset, study design and genotyping method did not influence the associations obviously.

**Guidelines for future research:** Future studies should use standardized unbiased genotyping methods and homogeneous cancer patients and well-matched controls and include multiethnic groups.

**Disclosure of funding source:** No funding supported this study.
